# Centrin-POC5 inner scaffold provides distal centriole integrity for sperm flagellar assembly

**DOI:** 10.1126/sciadv.aea4045

**Published:** 2025-12-03

**Authors:** Yutaka Takeda, Eriko Kajikawa, Jingwen Wang, Morié Ishida, Manfred Alsheimer, Hiroki Shibuya

**Affiliations:** ^1^ Laboratory for Gametogenesis, RIKEN Center for Biosystems Dynamics Research (BDR), Chuo-ku, Kobe, Hyogo 650-0047, Japan.; ^2^ Graduate School of Science, Osaka University, 1-1 Machikaneyama, Toyonaka, Osaka 560-0043, Japan.; ^3^ Department of Cell and Developmental Biology, Biocenter, University of Würzburg, 97074 Würzburg, Germany.

## Abstract

Centrioles undergo marked transformations during spermatogenesis that are essential for sperm motility and male fertility. Despite their importance, the molecular mechanisms and ultrastructural dynamics underlying these transformations remain largely unknown. Here, we apply ultrastructure expansion microscopy and reveal previously unrecognized centriolar architectural changes in mouse male germ cells, including geometry switching between the two centrioles and stage-specific removal of distal tip proteins such as centrin and SFI1. We further identify the centrin-POC5 inner scaffold as a key structure selectively augmented at the distal centriole, which directly forms and anchors the flagellum. Functional analyses of *Poc5* knockout mice demonstrate that this inner scaffold is essential for distal centriole integrity and flagellar assembly in spermatids but dispensable in somatic cells and spermatocytes. Our findings provide a spatiotemporal molecular atlas of centriole remodeling during spermatogenesis and uncover the critical physiological role of the centriolar centrin-POC5 inner scaffold in mammalian reproduction.

## Teaser

Expansion microscopy illuminates centriole dynamics during sperm development.

## INTRODUCTION

The centriole is a ubiquitous macromolecular structure that is crucial for faithful cell division, signal transduction, and sperm motility by organizing the centrosomes, cilia, and flagella (*1*–*4*). The mature mammalian centriole, with its cylindrical shape measuring about 450 nm in length and 200 nm in width, consists of triplet microtubules arranged in a ninefold symmetry along with various structural elements, including the inner scaffold, the distal tip, the distal cap, the luminal distal ring, the appendages, and the A-C linkers (*5*, *6*).

Canonical centrioles in somatic cells undergo one-to-one duplication once per cell cycle (*7*–*10*). During the G_1_ phase, somatic cells normally have two mother centrioles (*7*, *9*, *10*). A single new centriole, termed the procentriole, forms perpendicular to the lateral wall of each mother centriole in the S phase and grows in length and width during the G_2_ phase (*7*–*10*). In the M phase, each centriole pair matures into a mitotic centrosome by expanding its pericentriolar materials to establish a bipolar spindle, and, concurrent with chromosome segregation, the two centrosomes are equally divided into daughter cells (*7*, *9*–*11*). In addition, in the quiescent state, the oldest mother centriole grows a primary cilium that functions as a key coordinator of signaling pathways (*7*, *12*, *13*). While the regulation of centrioles in somatic cells has been extensively studied as described above, their regulation in male germ cells, despite their essentiality for flagellar assembly, is poorly understood.

During sperm development, the two canonical centrioles undergo specific transformations to become the distal centriole (DC) and the proximal centriole (PC) within the spermatid (*14*). The DC in mammalian sperm adopts a fan-like shape, optimized for its function as the flagellum base, whereas canonical centrioles exhibit a cylindrical morphology (*15*–*17*). The PC attaches laterally to the nucleus and develops a specialized structure, the centriolar adjunct, at its distal end (*15*, *18*, *19*). These observations suggest that centriolar structural elements undergo dynamic rearrangements through germ cell–specific molecular regulations. However, the ultrastructural changes that occur in centrioles during these transformations and the underlying molecular mechanisms behind them remain to be elucidated.

Recently, ultrastructure expansion microscopy (U-ExM) has emerged as a powerful technique for the nanoscale molecular mapping of proteins (*20*). Unlike other super-resolution techniques, U-ExM is based on isotropic sample expansion in all dimensions, enabling high-throughput super-resolution imaging with standard microscopes and reagents (*6*, *20*). The application of U-ExM to centrioles in male germ cells is unprecedented and offers the potential to visualize the intricate changes in the centriolar architecture during these structural transformations.

Here, we present a U-ExM method tailored for mouse male germ cells and demonstrate previously unrecognized centriole transformations during spermatogenesis. Our approach reveals unexpected remodeling, including geometry switching between the mother centriole and procentriole and the removal of distal tip proteins, which coincides with the structural transformations that occur in the development from spermatocytes to spermatids. Notably, we identify a unique augmentation of the centriolar centrin-POC5 inner scaffold at the DC. Through in-depth phenotypic analyses of *Poc5* knockout (KO) mice, we show that this reinforced inner scaffold serves as a structural basis of DCs and is indispensable for flagellar assembly. Collectively, our findings redefine the transformations of centriolar architecture during spermatogenesis, unveiling a critical mechanism that ensures reproductive success.

## RESULTS

### U-ExM reveals geometry switching between the two centrioles during spermatogenesis

**Fig. 1.**
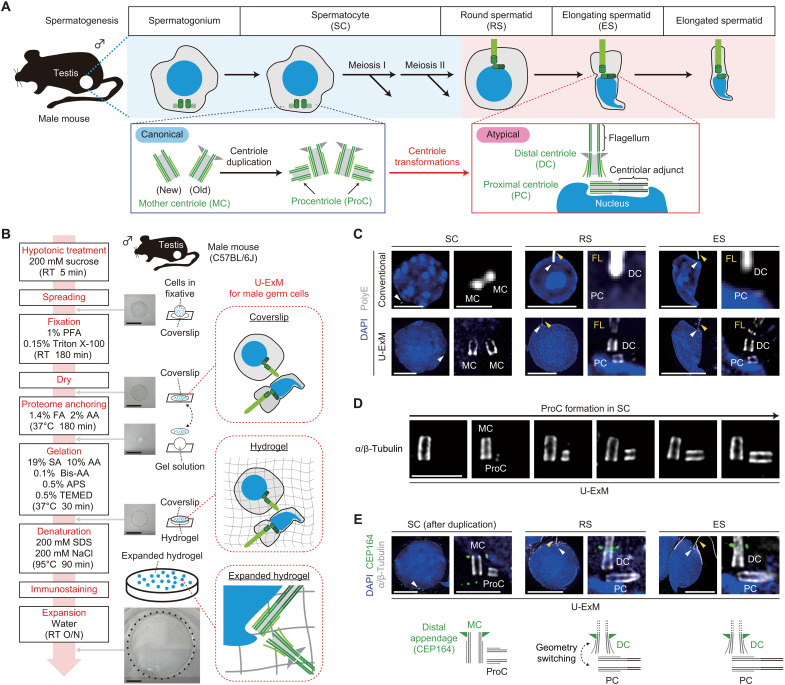
U-ExM reveals geometry switching between the two centrioles during spermatogenesis. (**A**) Schematic showing centriole dynamics during mouse spermatogenesis. (**B**) Schematic of U-ExM for mouse male germ cells. RT, room temperature; PFA, paraformaldehyde; FA, formaldehyde; AA, acrylamide; SA, sodium acrylate; Bis-AA, bis-acrylamide; O/N, overnight. Scale bars, 1 cm. (**C**) Conventional IF and U-ExM images of spermatocytes (SCs), round spermatids (RSs), and elongating spermatids (ESs) from wild-type (WT) male mice. Arrowheads: centrioles (white) and flagella (yellow). FL, flagellum. Scale bars, 10 and 1 μm. (**D**) U-ExM images of SCs from WT male mice. Images of different SCs were arranged according to the length of the procentrioles (ProCs). Scale bar, 1 μm. (**E**) U-ExM images of SC, RS, and ES from WT male mice. Arrowheads: centrioles (white) and flagella (yellow). Scale bars, 10 and 1 μm.

During the differentiation from spermatocytes to spermatids, canonical centrioles specifically transform into the DC and the PC (Fig. 1A) (*14*). The DC serves as the basal body of the flagellum, which develops into the sperm tail (*15*–*17*), while the PC attaches to the nucleus and gives rise to a specialized structure known as the centriolar adjunct (*15*, *18*, *19*). These structural transformations of centrioles in spermatids are crucial for sperm development and successful fertilization. However, the ultrastructural changes that occur in centrioles and the underlying molecular mechanisms behind these transformations remain poorly understood. Because of the small size of the centriole ultrastructures, visualization using conventional immunofluorescence (IF) techniques is challenging. Therefore, we developed a U-ExM protocol specifically optimized for mouse male germ cells by modifying the established method for cultured mammalian cells (Fig. 1B) (*6*, *20*). As male germ cells are nonadherent cells isolated from the tissue, we gently fixed and dried the testis cell suspension onto coverslips to ensure stable immobilization. In addition, we applied a hypotonic treatment before fixation to remove excess cytoplasm, which improved permeability and resolution in IF. The subsequent steps—proteome anchoring, hydrogelation, denaturation, immunostaining, and expansion—followed the standard U-ExM procedure. Using this modified U-ExM method, the centriolar microtubule structure in male germ cells—observed as a single focus by conventional IF—was observed with its original barrel-like morphology (Fig. 1C). Furthermore, this technique enabled the distinguishment of the centriolar microtubules from the flagellar microtubules in spermatids (Fig. 1C).

In canonical centriole duplication within somatic cells, the procentriole assembles perpendicular to the lateral wall of the preexisting mother centriole (*7*, *9*). We found that the same geometrical relationship applies to centriole duplication in spermatocytes (Fig. 1D and fig. S1A). However, in spermatid development, the centrioles’ geometrical relationship appears to be inverted; the DC, the putatively older centriole analogous to the basal body of primary cilia in somatic cells, attaches to the lateral wall of the PC (Fig. 1E). In all the spermatids we examined, centrosomal protein 164 (CEP164), a marker of distal appendages that are specifically present on fully mature centrioles (*21*, *22*), was exclusively detected on DCs but not on PCs, mirroring its localization on mother centrioles in spermatocytes (Fig. 1E). In previous studies, the origins of the DC and the PC in spermatids, specifically whether they derive from the mother centriole or the procentriole, remain a subject of debate (*23*). Some researchers argue that DCs derive from mother centrioles because single cilia in somatic cells arise from the older centrioles (*24*, *25*). Conversely, others claim a procentriole origin because the DC base attaches orthogonally to the PC wall, similar to how a procentriole attaches to a mother centriole (*19*, *23*). Our results unequivocally demonstrate that mother centrioles and procentrioles in spermatocytes transform into DCs and PCs in spermatids, respectively, and these transformations involve geometry switching, characterized by an inversion of their spatial relationship during spermatocyte-to-spermatid development. Furthermore, a small subset of early round spermatids was undergoing geometry switching, during which the orthogonal relationship between the two centrioles was being dissolved (fig. S1B). Collectively, we successfully established a U-ExM method for mouse male germ cells and have addressed the longstanding question regarding the origins of the DC and the PC in spermatids.

### Selective removal of distal tip proteins occurs during centriole transformations

**Fig. 2.**
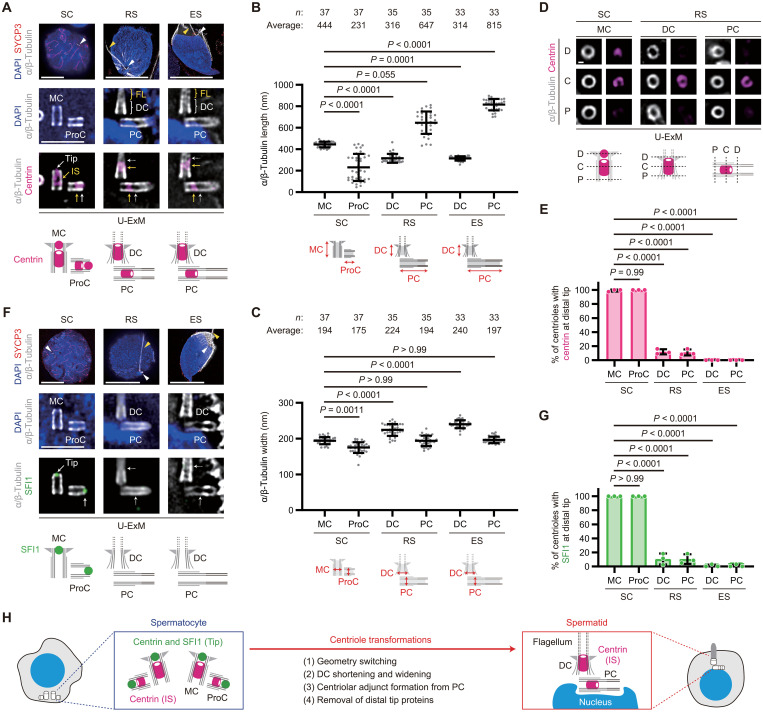
Selective removal of distal tip proteins occurs during centriole transformations. (**A**) U-ExM images of SC, RS, and ES from WT male mice. Arrowheads: centrioles (white) and flagella (yellow). Tip, distal tip; IS, inner scaffold. Scale bars, 10 and 1 μm. (**B** and **C**) Quantification of the centriole length (B) and width (C) based on the α/β-tubulin signal in U-ExM images. (**D**) U-ExM images of SC and RSs from WT male mice. Cross-sectioned centrioles are shown. D, distal region; C, central region; P, proximal region. Scale bar, 100 nm. (**E**) Quantification of the frequency of centrioles with centrin signals at the distal tip in U-ExM images. *N* = 3 independent experiments with >30 cells each. (**F**) U-ExM images of SC, RS, and ES from WT male mice. Arrowheads: centrioles (white) and flagella (yellow). Scale bars, 10 and 1 μm. (**G**) Quantification of the frequency of centrioles with SFI1 signal at the distal tip in U-ExM images. *N* = 3 independent experiments with >30 cells each. (**H**) Schematic summarizing the observations. Data are presented as the mean ± SD. *P* values were calculated by one-way analysis of variance (ANOVA) with Dunn’s multiple-comparison test [(B) and (C)] or Dunnett’s multiple-comparison test [(E) and (G)].

Using the U-ExM method for male germ cells, we visualized the molecular architecture of centrioles by immunostaining with antibodies against α/β-tubulin and centrin, a canonical centriolar protein (Fig. 2A). We initially quantified the length and width of the centriolar microtubule structure at each developmental stage of male germ cells based on the α/β-tubulin signals. In spermatocytes, the average length (444 nm) and width (194 nm) of the mother centrioles were consistent with those reported for somatic cells in previous studies (Fig. 2, B and C) (*6*). Regarding procentrioles, the substantial standard deviations in their length and width (Fig. 2, B and C) reflect their gradual elongation and blooming processes during spermatocyte development (Fig. 1D), as previously described for procentrioles in somatic cells (*6*). These results indicate that mother centrioles and procentrioles in spermatocytes are structurally similar to those in somatic cells. In contrast, DCs in round and elongating spermatids exhibited a shorter length and a greater width compared to mother centrioles in spermatocytes (Fig. 2, B and C). PCs showed a substantial increase in average length, indicating centriolar adjunct formation, while their average width remained comparable to that of mother centrioles in spermatocytes (Fig. 2, B and C). These structural transformations of DCs and PCs were largely consistent with prior observations using transmission electron microscopy (TEM) or super-resolution microscopy (*15*–*19*). Together, DCs in spermatids undergo shortening and widening, likely in preparation for flagellar assembly, while PCs remain unchanged except for the formation of the centriolar adjunct.

Centrin localization in spermatocytes, as visualized by the U-ExM method, revealed two distinct populations—one at the distal tip and one at the inner scaffold (Fig. 2, A and D and fig. S1C)—in agreement with the reported bimodal centrin localization in somatic cells (*6*, *26*, *27*). The distal tip centrin signal was lost in round and elongating spermatids, while the inner scaffold centrin signal remained (Fig. 2, A, D, and E, and fig. S1, C and D). The removal of distal tip centrin occurred at the early stage of round spermatid development, as the early round spermatids in the process of geometry switching retained centrin signals at the distal tips (fig. S1B), reflecting the transitional state of centriole transformations.

Given that centrin recruitment to the distal tip is SFI1 dependent in human cells, where SFI1 is also localized to the distal tip (*26*), we investigated SFI1 localization in male germ cells. In spermatocytes, SFI1 was specifically localized to the distal tip of both mother centrioles and procentrioles, whereas it was absent from both DCs and PCs in round and elongating spermatids (Fig. 2, F and G). These data suggest that the removal of SFI1 leads to the loss of the distal tip centrin population during centriole transformations. Notably, both centrin and SFI1 remained at the distal tip of mother centrioles during primary cilium formation in mouse embryonic fibroblasts (MEFs) (fig. S1, E to I), indicating that the removal of distal tip proteins occurred for flagellar assembly in male germ cells but not for ciliary assembly in somatic cells. The distal cap protein CP110 (*6*, *28*) also detached from both DCs and PCs in most spermatids (fig. S2A), while the luminal distal ring protein C2CD3 (*6*, *29*), which functions as an architectural hub of the distal end (*29*), was retained (fig. S2B). This indicates that the distal tip and cap are selectively removed from the distal end during centriole transformations. Together, our U-ExM technique successfully delineated the following centriole dynamics in male germ cells (Fig. 2H). (i) The spatial relationship between the two centrioles is inverted (geometry switching). (ii) DCs in spermatids undergo shortening and widening. (iii) PCs in spermatids initiate centriolar adjunct formation. (iv) The distal tip proteins are removed from DCs and PCs in spermatids.

### Centrin-POC5 inner scaffold is augmented in the DC during centriole transformations

**Fig. 3.**
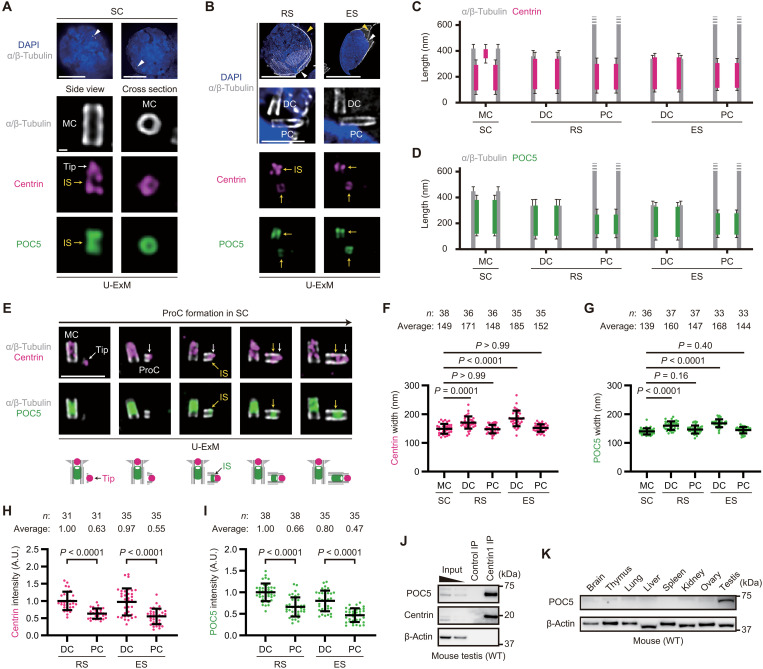
Centrin-POC5 inner scaffold is augmented in the DC during centriole transformations. (**A**) U-ExM images of SCs from WT male mice. Arrowheads: centrioles. Scale bars, 10 and 100 nm. (**B**) U-ExM images of RS and ES from WT male mice. Arrowheads: centrioles (white) and flagella (yellow). Scale bars, 10 and 1 μm. (**C** and **D**) Schematic showing the longitudinal positions of centrin (magenta; C) and POC5 (green; D) relative to the α/β-tubulin signal (gray) calculated from >30 centrioles in U-ExM images. (**E**) U-ExM images of SCs from WT male mice. Images of different SCs were arranged according to the length of the ProCs. Scale bar, 1 μm. (**F** and **G**) Quantification of the widths of centrin (F) and POC5 (G) signals at centrioles in U-ExM images. (**H** and **I**) Quantification of centrin (H) and POC5 (I) intensity at centrioles in U-ExM images. A.U., arbitrary units. (**J**) Immunoblotting (IB) images of immunoprecipitated lysates from WT mouse testis extracts. (**K**) IB images of extracts from multiple tissues of WT mice. Data are presented as the mean ± SD. *P* values were calculated by one-way ANOVA with Dunn’s multiple-comparison test [(F) and (G)] or Mann-Whitney *U* test [(H) and (I)].

To characterize the properties of the centriolar inner scaffold during centriole transformations, we investigated the localization of centrin together with POC5, a protein essential for centrin recruitment to the inner scaffold, but not the distal tip (Fig. 3, A and B) (*26*). Spatiotemporal quantification of centrin and POC5 localization demonstrated their persistent colocalization at the inner scaffold throughout spermatogenesis (Fig. 3, C and D). Analysis of their recruitment timing into newly formed procentrioles in spermatocytes revealed that while the distal tip centrin is present from the early stage of procentriole formation, centrin and POC5 at the inner scaffold are recruited later in a synchronous manner (Fig. 3E). These results suggest that centrin at the centriolar inner scaffold is regulated synchronously with POC5 and forms a distinct centrin-POC5 complex, separate from the distal tip centrin population in male germ cells.

Notably, there were differences in the localization patterns of centrin and POC5 between the DC and the PC in spermatids (Fig. 3B). The average lengths of centrin and POC5 distributions at the inner scaffold of DCs were greater than those of PCs (Fig. 3, C and D). Furthermore, as the DCs themselves widened in spermatids (Fig. 2C), the average widths of centrin and POC5 signals at the inner scaffold increased in DCs but not in PCs (Fig. 3, F and G). Regarding signal intensity, a proxy measure of protein amount, centrin and POC5 exhibited significantly higher values in DCs compared to those in PCs (Fig. 3, H and I), indicating a quantitative asymmetry of centrin and POC5 between the DC and the PC. A similar trend was observed in U-ExM observations of other inner scaffold components such as POC1A and POC1B (*6*, *27*, *30*–*32*), although the degree varied (fig. S3, A to H). Furthermore, the DC-PC asymmetry of centrin and POC5 was also observed in signal intensity quantifications using conventional IF (fig. S3, I to K), confirming that it was not an artifact of the U-ExM technique.

Given that human POC5 directly interacts with human centrin (*33*), we performed immunoprecipitation (IP) of mouse centrin paralogs (centrin1 and centrin2) in mouse testis extracts and successfully detected their interactions with mouse POC5 (Fig. 3J and fig. S4A). In addition, AlphaFold-Multimer predictions of protein complex structures (*34*, *35*) indicated that POC5 directly binds to all centrin paralogs in both humans and mice, where the EF hands of centrin molecules wrap around the longest coiled coil of POC5 (fig. S4, B and C). Of note, the expression of POC5 was substantially up-regulated in testes compared to other somatic tissues (Fig. 3K), suggesting the importance of POC5 predominately in the male reproductive organ. Collectively, the testis-enriched protein POC5 directly interacts with centrin in male germ cells, and their localization at the inner scaffold is specifically augmented in DCs during spermatid development.

### POC5 is required for spermatogenesis and male fertility in mice

**Fig. 4.**
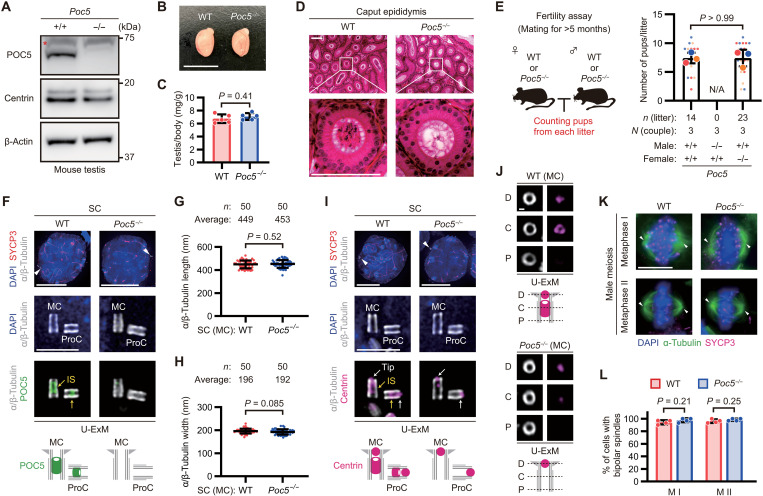
POC5 is required for male fertility but not for centriole integrity and function in SCs. (**A**) IB images of testis extracts. Asterisk: nonspecific band. (**B**) Images of testes from 12-week-old male mice. Scale bar, 1 cm. (**C**) Quantification of the testis weight normalized to body weight. *N* = 5 mice. (**D**) Images of caput epididymis sections from 12-week-old male mice. The sections were stained with hematoxylin and eosin. Scale bars, 100 μm. (**E**) Quantification of the number of pups per litter. N/A, not applicable. (**F**) U-ExM images of SCs from male mice. Arrowheads: centrioles. Scale bars, 10 and 1 μm. (**G** and **H**) Quantification of the mother centriole (MC) length (G) and width (H) based on the α/β-tubulin signal in U-ExM images of SCs. (**I**) U-ExM images of SCs from male mice. Arrowheads: centrioles. Scale bars, 10 and 1 μm. (**J**) U-ExM images of SCs from male mice. Cross-sectioned MCs are shown. Scale bar, 100 nm. (**K**) IF images of SCs from male mice. The metaphase cells were classified into metaphase I and II based on the SYCP3 signal. The representative cells were prepared by the squash technique. Arrowheads: spindle poles. Scale bar, 10 μm. (**L**) Quantification of the frequency of metaphase cells with bipolar spindles. *N* = 4 independent experiments with ≥30 cells each. M, metaphase. Data are presented as the mean ± SD. *P* values were calculated by two-tailed unpaired Student’s *t* test [(C), (E), and (L)] or Mann-Whitney *U* test [(G) and (H)].

To elucidate the role of the centrin-POC5 inner scaffold in the structural transformations of centrioles in male germ cells, we generated *Poc5* KO mice by CRISPR-Cas9 genome editing (fig. S5, A and B). We targeted *Poc5* because centrin has multiple redundant paralogs, making simultaneous abrogation of all centrin technically challenging. Western blot analysis confirmed the loss of POC5 expression in the KO testis samples (Fig. 4A). *Poc5* KO mice were born at Mendelian ratios (fig. S5C) and remained viable with no detectable developmental defects, including no difference in testis size (Fig. 4, B and C) or organogenesis (fig. S5D), indicating that POC5 is dispensable for mouse development and viability. However, epididymis sections from *Poc5* KO mice showed the absence of spermatozoa (Fig. 4D). This led to complete male infertility, while female was unaffected (Fig. 4E). These results demonstrate that POC5 is essential for sperm development and male fertility in mice yet dispensable for overall mouse development and viability.

### POC5 is dispensable for centriole integrity and function in spermatocytes and in somatic cells

To investigate the role of the centriolar centrin-POC5 inner scaffold during spermatogenesis, we analyzed the structure and function of centrioles within *Poc5* KO male germ cells. We initially examined the structural integrity of centrioles in spermatocytes before centriole transformations. Even with the complete abrogation of POC5 localization at the centriolar inner scaffold, the centriolar microtubule structure, including the perpendicular attachment of procentriole to the mother centriole, appeared intact in *Poc5* KO spermatocytes (Fig. 4F). Quantifications showed that the average length and width of the mother centrioles were not altered (Fig. 4, G and H), indicating that POC5 is dispensable for the structural integrity of centrioles in spermatocytes. We then compared the localization of several centriolar proteins between wild-type (WT) and *Poc5* KO spermatocytes. Centrin was completely lost from the inner scaffolds but remained distinctly localized at the distal tips in *Poc5* KO spermatocytes (Fig. 4, I and J, and fig. S6, A to C), likely in a SFI1-dependent manner, consistent with previous findings in cultured human cells (*26*). We confirmed that SFI1 was recruited normally to the distal tips even in *Poc5* KO spermatocytes (fig. S6, D and E). The recruitment of CP110 to the distal caps of centrioles was also unaffected in *Poc5* KO spermatocytes (fig. S6, F and G). The localization of POC1A and POC1B was unaffected in *Poc5* KO spermatocytes (fig. S6, H to J), consistent with a previous report on somatic *POC5* KO cells, in which POC1A and POC1B are recruited to the inner scaffold as upstream factors of centrin and POC5 (*27*). Together, POC5 depletion abrogates the localization of centrin specifically at the centriolar inner scaffold without affecting the overall structural integrity of centrioles in spermatocytes.

Homologous chromosomes undergo synapsis during meiotic prophase I, and the progression of synapsis defines the substages of prophase I (*36*, *37*). Staining of synaptonemal complex protein 1 (SYCP1), which localizes to the synapsed chromosomal axes, demonstrated that *Poc5* KO did not affect homologous synapsis and thus did not influence prophase I progression (fig. S6, K and L). After prophase I, spermatocytes undergo two successive rounds of cell division, where equal chromosome segregation between daughter cells is ensured by bipolar spindles that are formed in metaphase I and II (*25*, *38*). The formation and maintenance of the bipolar spindle depend on the centrosome, with the centrioles as its core structures (*11*, *25*). To assess the requirement for POC5 in centriole function for meiotic spindle formation and maintenance, we compared the morphology of meiotic spindles. Almost all *Poc5* KO spermatocytes established bipolar spindles comparable to WT in both metaphase I and II (Fig. 4, K and L). These results demonstrate that the centrin-POC5 inner scaffold is not required for centriole integrity and function in spermatocytes.

Notably, analyses of *Poc5* KO MEFs revealed similar results to those observed in spermatocytes. Despite the complete depletion of POC5 (fig. S7, A and B) and centrin (fig. S7, C and D) from the inner scaffold, the structural integrity of centrioles remained unaffected, including their length (fig. S7E) and width (fig. S7F). Furthermore, the recruitment of centrin (fig. S7, C and D), SFI1 (fig. S7, G and H), and CP110 (fig. S7, I and J) to the centriolar distal end was unaffected in the absence of POC5. *Poc5* KO MEFs properly maintained the number of centrioles during the mitotic cell cycle (fig. S7, K and L), and they matured into centrosomes (fig. S7, K and M), successfully establishing bipolar mitotic spindles (fig. S7, N and O). These results show that the centrin-POC5 inner scaffold is also dispensable for centriole integrity and function within somatic cells.

### POC5 ensures the structural integrity of DCs for flagellar assembly in spermatids

**Fig. 5.**
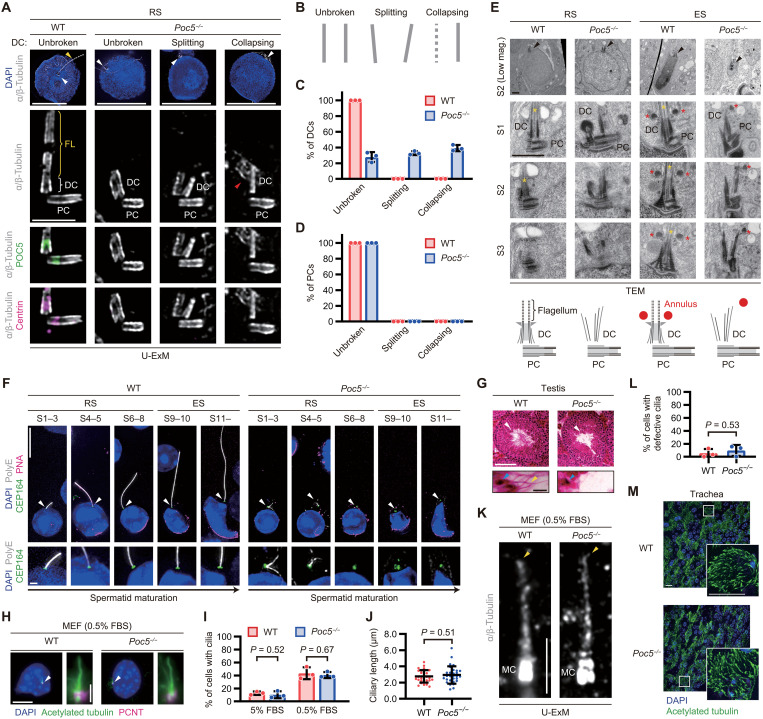
POC5 ensures the structural integrity of DCs for flagellar assembly in spermatids. (**A**) U-ExM images of RSs from male mice. Arrowheads: centrioles (white), flagellum (yellow), and missing centriole wall (red). Scale bars, 10 and 1 μm. (**B**) Schematic showing the classification of centriole structures. (**C** and **D**) Quantification of the frequency of DCs (C) and PCs (D) in U-ExM images of RSs. *N* = 3 independent experiments with >30 cells each. (**E**) TEM images of RSs and ESs from male mice. Three serial sections. Arrowheads: centrioles; asterisks: flagella (yellow) and annuli (red). mag., magnification. Scale bars, 1 μm. (**F**) IF images of RSs and ESs from male mice. Arrowheads: centrioles. Scale bars, 10 and 1 μm. (**G**) Images of 12-week-old male mouse testis sections stained with hematoxylin and eosin. Arrowheads: magnified spermatids (white), heads of spermatids (cyan), and flagellum (yellow). Scale bars, 100 and 10 μm. (**H**) IF images of serum-starved [0.5% fetal bovine serum (FBS)] MEFs. Arrowheads: primary cilia. Scale bars, 10 and 1 μm. (**I**) Quantification of the frequency of cells with primary cilia. *N* = 3 independent experiments with >100 cells each. (**J**) Quantification of the length of primary cilia. *n* > 30 cells. (**K**) U-ExM images of serum-starved (0.5% FBS) MEFs. Arrowheads: distal ends of primary cilia. Scale bar, 1 μm. (**L**) Quantification of the frequency of cells with defective primary cilia in U-ExM images. *N* = 3 independent experiments with >30 cells each. (**M**) IF images of mouse tracheae. Scale bars, 10 μm. Data are presented as the mean ± SD. *P* values were calculated by two-tailed unpaired Student’s *t* test [(I) and (L)] or Mann-Whitney *U* test (J).

To investigate the requirement of the centrin-POC5 inner scaffold for centriole transformations in male germ cells, we analyzed the structural integrity of both DCs and PCs in *Poc5* KO spermatids. The localization of POC5 and centrin was abolished in both DCs and PCs in *Poc5* KO round spermatids (Fig. 5A), which was further confirmed by immunostaining of seminiferous tubules (fig. S8). DCs exhibited substantial structural abnormalities in *Poc5* KO round spermatids, including splitting, where the microtubule wall of the DC was no longer parallel but instead split outward, and collapsing, where the microtubule wall was disintegrated (Fig. 5, A to C), while PCs appeared structurally normal (Fig. 5, A, B, and D). In *Poc5* KO round spermatids, other inner scaffold proteins, POC1A and POC1B, underwent structural disruption that coincided with the breakdown of DCs, whereas these proteins exhibited no localization defects at PCs (fig. S9, A to D). Furthermore, the flagellar microtubules extending from DCs, which were readily observed in WT round spermatids, were absent in most *Poc5* KO round spermatids (Fig. 5A), suggesting that these structural abnormalities in DCs impede flagellar assembly. TEM of centrioles in *Poc5* KO round spermatids further demonstrated that DCs were dispersed and failed to form flagella, whereas PCs remained structurally normal (Fig. 5E). In addition, in later stages of elongating spermatids, the annulus surrounding the flagellar microtubules (*39*, *40*) was distorted in *Poc5* KO spermatids, likely due to the collapse of the DC and the flagellum (Fig. 5E).

We then investigated the stepwise flagellar assembly in WT and *Poc5* KO spermatids, using acrosome staining with peanut agglutinin (PNA) as a marker for spermatid development (Fig. 5F) (*41*–*43*). In WT spermatids, flagella began to emanate from the CEP164-stained DCs at steps 1 to 3 and progressively elongated as spermatid maturation proceeded (Fig. 5F). In contrast, *Poc5* KO spermatids exhibited frayed or disorganized flagellum-like structures at steps 1 to 3, and these gradually degenerated in the subsequent stages (Fig. 5F). Moreover, testis sections from *Poc5* KO mice revealed minimal flagellar assembly (Fig. 5G), which was consistent with our U-ExM (Fig. 5A), TEM (Fig. 5E), and IF (Fig. 5F) analyses of isolated spermatids. Collectively, *Poc5* KO spermatids displayed severe defects, including DC structural abnormalities, annulus distortion, and flagellar assembly failure, suggesting that the centrin-POC5 inner scaffold is essential for the structural transformations of DCs and therefore is necessary for the assembly of flagella during spermatogenesis.

For signal transduction or fluid flow generation, centrioles within some types of somatic cells can form cilia that share a structural similarity with flagella (*7*, *12*, *13*). We next sought to determine whether the centriolar centrin-POC5 inner scaffold is specifically required for flagellar assembly in male germ cells or more generally required for ciliary assembly in somatic cells. To this end, we analyzed ciliary assembly in somatic cells from WT and *Poc5* KO mice. First, we analyzed primary cilium formation in MEFs under conditions of serum starvation [0.5% fetal bovine serum (FBS)] (Fig. 5H). The percentage of ciliated cells (Fig. 5I) and the average length of the primary cilia (Fig. 5J) showed no significant differences between the serum-starved WT and *Poc5* KO MEFs. Furthermore, imaging of primary cilia with the U-ExM method revealed no structural defects in *Poc5* KO MEFs (Fig. 5, K and L, and fig. S10, A and B). Subsequently, to assess the requirement of POC5 for ciliary assembly at the tissue level, we examined ciliated epithelial cells in the trachea and confirmed the normal motile cilium formation even in *Poc5* KO mice (Fig. 5M). Furthermore, *Poc5* KO mice exhibited no defects in left-right axis determination (fig. S10C), which requires both a unidirectional flow generated by motile cilia and the sensing of this flow by primary cilia in the mouse embryo (*44*–*47*). These results demonstrate that the centriolar centrin-POC5 inner scaffold is required for flagellar assembly in male germ cells but not for ciliary assembly in somatic cells.

## DISCUSSION

**Fig. 6.**
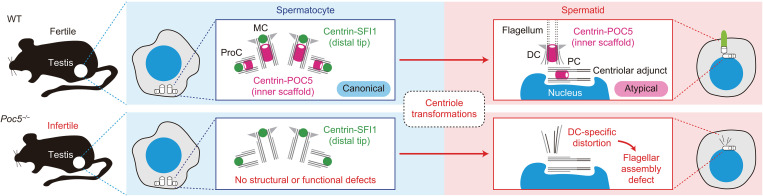
Centrin-POC5 inner scaffold provides DC integrity for sperm flagellar assembly. Schematic summarizing this study.

In spermatids, centrioles undergo structural transformations into the two specialized centrioles, namely the DC and the PC (*14*). This event is crucial for flagellar assembly, sperm motility, and reproductive success, but the ultrastructural changes within centrioles and the underlying molecular mechanisms behind these transformations were poorly understood. In this study, we used the U-ExM method in mouse male germ cells to visualize the molecular architecture of centrioles during these transformations, and we revealed geometry switching between the two centrioles and the removal of distal tip proteins in spermatids. Furthermore, we demonstrated the augmentation of the centriolar centrin-POC5 inner scaffold at the DC and its specific necessity for DC integrity, flagellar assembly, and male fertility throughout the phenotypic analyses of *Poc5* KO mice. Collectively, we propose that the primary role of the centriolar centrin-POC5 inner scaffold is to provide a structural basis for the sperm flagellum by transforming the canonical centriole into the DC (Fig. 6).

The removal of the distal tip proteins centrin and SFI1 from centrioles has not been previously reported in either cultured cells or in vivo contexts, and its functional significance remains enigmatic. In human somatic cells, RNA interference–mediated depletion of SFI1 results in structural distortions at the distal ends of centrioles (*26*), suggesting that the centrin-SFI1 complex is crucial for maintaining the structural rigidity of the centriolar distal end. We hypothesize that the DC adaptively discards its distal tip structure to acquire flexibility, rather than rigidity, for anchoring the motile flagellum. This hypothesis aligns with our findings that these distal tip proteins are present in the mother centrioles of MEFs that have nonmotile primary cilia. By contrast, the removal of distal tip proteins might be necessary for the assembly of the centriolar adjunct at the distal end of the PC. To test these hypotheses, identifying the regulators that promote the removal of distal tip proteins in spermatids is critical.

The centrin-POC5 centriolar inner scaffold is specifically required for the structural integrity of the DC and is therefore crucial for flagellar assembly. Consequently, *Poc5* KO mice exhibited azoospermia and male infertility. In contrast, the centrin-POC5 inner scaffold appears to be dispensable for the structural integrity and function of centrioles in somatic cells as evidenced by the fact that *Poc5* KO mice were born at a Mendelian ratio, developed normally, and had no detectable centriolar or ciliary defects. The unique requirement of the centrin-POC5 inner scaffold in the DC during spermatid development might be explained by the atypical structural composition of the DC. Previous studies suggest that the DC lacks the A-C linkers connecting the triplet microtubules (*23*, *48*). In the other centriole types, including canonical centrioles in somatic cells and spermatocytes and PCs in spermatids, the inner scaffold and the A-C linkers may cooperatively provide the structural integrity of centrioles. Therefore, even when the inner scaffold is disrupted by POC5 depletion, the A-C linkers can sufficiently maintain the overall structure of centrioles. In contrast, the DC, lacking the A-C linkers, probably preserves its structural integrity by augmenting the inner scaffold instead. This idea is supported by the fact that depletion of A-C linker components in cultured human cells also enhanced the localization of POC5 at the centriolar inner scaffold (*49*). Thus, the DC cannot retain its overall structure when the inner scaffold collapses. Consistent with this, canonical centrioles within cultured human cells were substantially disrupted upon simultaneous depletion of WDR67, an A-C linker component, and POC5 (*49*).

Our U-ExM protocol successfully visualized the unique molecular architecture of centrioles in male germ cells, which conventional IF could not resolve. Further application of this technique to other structural elements of centrioles (*6*) will reveal distinct characteristics of centrioles in male germ cells, thus advancing our understanding of centriole dynamics during sperm development. Moreover, the U-ExM approach enabled the super-resolution imaging of other germ cell–specific macromolecular structures, including the synaptonemal complex (Fig. 2A), the manchette (Fig. 1E), and the flagellum (Fig. 1, C and E) (*36*, *37*, *50*–*55*). This technique will lead to unexplored discoveries not only in centriole biology but also in various other fields related to spermatogenesis. Overall, our findings provide fundamental insights into the structural transformations of centrioles specific to male germ cells, which are essential for flagellar assembly, successful fertilization, and thus our species survival.

## MATERIALS AND METHODS

### Mice

All animal experiments in this study were approved by the Institutional Animal Care and Use Committee at the RIKEN Kobe branch (IACUC; A2025-02). All mice were housed in a 12-hour light–and–12-hour dark cycle with free access to food and water. *Poc5* KO mice were generated in this study by CRISPR-Cas9 genome editing. Two guide RNAs (#1: 5′-GGAGGCTGAACTTTATCGAC[TGG]-3′, #2: 5′-AAACCAGATCTCGGACCTGA[AGG]-3′) were designed in exon 7 of *Poc5*. The mouse genotypes were confirmed with primers (forward: 5′-AGCCCTTGTGTTGACCTGAT-3′, reverse: 5′-ATTTCCACTGGGACATCCGC-3′). All WT and KO mice were congenic with the C57BL/6J background. Seven- to 20-week-old male mice were used to obtain male germ cells.

### Cell culture

MEFs were established from embryonic day 10.5 embryos from a *Poc5*^+/−^ mouse mating and then genotyped. The cells were cultured in Dulbecco’s modified Eagle’s medium (Nacalai Tesque, 08459-64) supplemented with 10% FBS (Biowest, S1780-500, lot: S15064S1780), 1 mM sodium pyruvate (Nacalai Tesque, 06977-34), penicillin (100 U/ml), and streptomycin (100 μg/ml; Nacalai Tesque, 09367-34) at 37°C in a 5% CO_2_ atmosphere.

### Antibodies

The following primary antibodies were used: rabbit polyclonal antibodies against POC5 [this study; 1:500 for IF, 1:1000 for immunoblotting (IB), and 1:250 for U-ExM], SFI1 (Proteintech, 13550-1-AP; 1:250 for U-ExM), POC1A (this study; 1:250 for U-ExM), POC1B (this study; 1:250 for U-ExM), centrin1 (this study; for IP), centrin2 (this study; for IP), CP110 (Proteintech, 12780-1-AP; 1:250 for U-ExM), CEP164 (Novus Biologicals, NBP1-81445, lot: 000012571; 1:500 for IF), CEP164 (Proteintech, 22227-1-AP, lot: 00045133; 1:250 for U-ExM), α-tubulin (MBL, PM054, lot: 011; 1:500 for IF), pericentrin (Abcam, ab4448; 1:1000 for IF), and SYCP1 (Abcam, ab15090, lot: GR3272415-4; 1:1000 for IF); chicken polyclonal antibody against SYCP3 [Hiroki Shibuya Lab (*56*); 1:3000 for IF and 1:1000 for U-ExM]; sheep polyclonal antibody against C2CD3 (R&D Systems, AF7348, lot: CFQX0122121; 1:125 for U-ExM); mouse monoclonal antibodies against centrin (Merck Millipore, 04-1624, clone: 20H5; 1:1000 for IF, 1:5000 for IB, and 1:500 for U-ExM), β-actin (Sigma-Aldrich, A2228-200UL, clone: AC-74; 1:2000 for IB), γ-tubulin (Abcam, ab27074, clone: TU-30; 1:500 for IF), Polyglutamylation modification (AdipoGen, AG-20B-0020-C100, clone: GT335; 1:1000 for IF), and acetylated tubulin (Sigma-Aldrich, T6793-100UL, clone: 6-11B-1; 1:1000 for IF); guinea pig monoclonal antibodies against α-tubulin (ABCD Antibodies, AA345; 1:250 for U-ExM) and β-tubulin (ABCD Antibodies, AA344; 1:250 for U-ExM); and rat monoclonal antibody against centrin2 [BioLegend, 698602, clone: W16110A; 1:200 for IF]. The following secondary antibodies were used: Alexa Fluor 488 donkey anti-mouse immunoglobulin G (IgG; H + L) (Thermo Fisher Scientific, A21202; 1:500 for IF), Alexa Fluor 488 donkey anti-rabbit IgG (H + L) (Thermo Fisher Scientific, A21206; 1:500 for IF), Alexa Fluor 488 goat anti-guinea pig IgG (H + L) (Thermo Fisher Scientific, A11073; 1:800 for U-ExM), Alexa Fluor 488 donkey anti-sheep IgG (H + L) (Thermo Fisher Scientific, A11015; 1:800 for U-ExM), Alexa Fluor 488 goat anti-chicken IgG (H + L) (Thermo Fisher Scientific, A32931; 1:500 for IF), Alexa Fluor 488 donkey anti-rat IgG (H + L) (Thermo Fisher Scientific, A21208; 1:500 for IF), Alexa Fluor 555 donkey anti-mouse IgG (H + L) (Thermo Fisher Scientific, A31570; 1:500 for IF and 1:800 for U-ExM), Alexa Fluor 555 donkey anti-rabbit IgG (H + L) (Thermo Fisher Scientific, A31572; 1:500 for IF and 1:800 for U-ExM), Alexa Fluor 555 goat anti-guinea pig IgG (H + L) (Thermo Fisher Scientific, A21435; 1:800 for U-ExM), Alexa Fluor 647 donkey anti-mouse IgG (H + L) (Thermo Fisher Scientific, A31571; 1:800 for U-ExM), Alexa Fluor 647 donkey anti-rabbit IgG (H + L) (Thermo Fisher Scientific, A31573; 1:800 for U-ExM), Alexa Fluor 647 donkey anti-chicken IgG (H + L) (Thermo Fisher Scientific, A78952; 1:500 for IF and 1:800 for U-ExM), horseradish peroxidase (HRP)–conjugated goat polyclonal antibodies against mouse IgG (Sigma-Aldrich, A4416-1ML; 1:5000 for IB), and HRP-conjugated goat polyclonal antibodies against rabbit IgG (Sigma-Aldrich, A0545-1ML; 1:5000 for IB).

### Antibody production

The antibodies against POC5, POC1A, and POC1B were generated by immunizing rabbits with recombinant proteins. cDNA encoding mouse POC5 (amino acids 359 to 558), mouse POC1A (amino acids 296 to 360), or mouse POC1B (amino acids 295 to 429) was cloned into the pET28c^+^ vector. The His-tagged recombinant proteins were expressed in BL21 (DE3) cells (Thermo Fisher Scientific, EC0114), solubilized in lysis buffer [600 mM NaCl, 30 mM imidazole, 20 mM tris-HCl (pH 7.5), and 0.1% Triton X-100], and purified with Ni–nitrilotriacetic acid resin (QIAGEN, 30230). The recombinant proteins were dialyzed in phosphate-buffered saline (PBS) and used to immunize rabbits. The antibodies against centrin1 and centrin2 were generated by immunizing rabbits with centrin1 (STFRKSNVASTSYKRKVG) or centrin2 (SNFKKTTMASSAQRKRMS) peptides. The polyclonal antibodies were purified on antigen-coupled CNBr-Activated Sepharose 4B beads (Cytiva, 17043001).

### Male germ cell spreading

Testes were dissected from male mice and were minced with flat-head forceps in PBS. The cell suspensions were washed with PBS several times and resuspended in hypotonic buffer [30 mM tris-HCl (pH 7.5), 17 mM trisodium citrate (pH 7.5), 5 mM EDTA (pH 8.0), and 50 mM sucrose] at room temperature for 10 min. The cells were centrifuged, and the pellets were resuspended in 200 mM sucrose at room temperature for 5 min. An equal volume of fixation buffer (1% paraformaldehyde and 0.1% Triton X-100 in PBS) was then added, and the cell suspensions were placed on slides (Epredia, J1800AMNZ; for conventional IF) or 12-mm round coverslips (Matsunami, C012001; for U-ExM). After fixation at room temperature for 3 hours under humid conditions, the cell suspensions on the slides and coverslips were air dried.

### Male germ cell squash

Testes were dissected from male mice, and seminiferous tubules were removed and placed on a glass slide, minced in a drop of fixation buffer (2% paraformaldehyde and 0.1% Triton X-100 in PBS), and incubated for 10 min. The coverslip was put on the glass slide, and the sample was frozen at −80°C until immunostaining.

### Frozen testis sections

Testes were dissected from male mice, embedded in Tissue-Tek O.C.T. Compound (Sakura Finetek, 4583) with disposable molds (Polysciences, 18985), and frozen at −80°C. The frozen blocks were subsequently sectioned at a thickness of 8 μm on a Cryostar NX70 (PHC) at −20°C. The serial frozen sections were fixed in either methanol (for 5 min at −20°C) or 4% paraformaldehyde (for 1 hour at room temperature) and then immunostained.

### Tracheal dissection

Tracheae were isolated from adult mice and cut longitudinally in ice-cold PBS. The fragmented tracheae were fixed in 4% paraformaldehyde for 30 min at 4°C, permeabilized by PBS containing 0.2% Triton X-100 for 30 min at 4°C, and then processed for immunostaining.

### Immunofluorescence

Cells on slides were blocked by 5% bovine serum albumin (BSA) in PBS for 30 min and then incubated with primary antibodies overnight at 4°C. After washing twice with PBS containing 0.1% Triton X-100 (PBS-X) for 5 min and once with PBS for 5 min, the cells were incubated with secondary antibodies, fluorescein isothiocyanate–labeled PNA (optional, 2 μg/ml; Sigma-Aldrich, L7381-1MG), Cy5-labeled PNA (optional, 1:1000; Vector Laboratories, VEC-CL-1075-1), and 4′,6-diamidino-2-phenylindole (DAPI) (1:1000; Wako, 045-30361) for 1 hour at room temperature. The cells were then washed twice with PBS-X for 5 min and once with PBS for 5 min in the dark and mounted in VECTASHIELD mounting medium (Vector Laboratories, H-1000).

### Ultrastructure expansion microscopy

U-ExM for mouse male germ cells and MEFs was performed according to the protocol for cultured human cells (*6*, *20*) with slight modifications. Mouse male germ cells spread and fixed on 12-mm round coverslips, or MEFs cultured on 12-mm round coverslips were incubated in proteome anchoring solution (1.4% formaldehyde and 2% acrylamide in PBS) for 3 hours at 37°C. The coverslips were then placed in gel solution [19% sodium acrylate, 0.1% bis-acrylamide, 10% acrylamide, 0.5% tetramethylethylenediamine (TEMED), and 0.5% ammonium persulfate] on ice for 5 min and then at 37°C for 30 min. After the gels had polymerized on the coverslips, they were boiled in denaturation buffer [50 mM tris-HCl (pH 9.0), 200 mM SDS, and 200 mM NaCl] at 95°C for 90 min. The gels were then transferred to water at room temperature for expansion. The expanded gels were measured in diameter for scale calculations and were cut into quarters. The gels were then incubated with primary antibodies diluted in 2% BSA in PBS for 3 hours at 37°C with shaking. After washing three times with PBS, the gels were incubated in 2% BSA in PBS with secondary antibodies and DAPI (1:1000) for 2.5 hours at 37°C with shaking. The immunostained gels were washed three times with PBS and then fully expanded in water overnight at room temperature.

### Fluorescence microscopy

For all fluorescence imaging, THUNDER Imaging Systems (Leica) with 63× and 100×/1.40 numerical aperture objective lens were used. The *Z* interval was set to 270 nm. For the representative U-ExM images, computational clearing and deconvolution (Lng LVCC) was performed using THUNDER Imaging Systems (Leica) with the following settings. Strategy: adaptive, refractive index: 1.33, mounting medium: Water, feature scale: 1000 nm (DAPI) or 339 nm (the other channels), strength: 92%, sensitivity: 1.0, cutoff: auto, regularization: 0.05, and smoothing: high. Unless otherwise noted, the representative images were generated by maximum intensity Z-projections using at least 11 steps. The representative U-ExM images showing enlarged views of centrioles in Fig. 5A and fig. S9 (A and B) were generated by maximum intensity Z-projections using five steps. All the representative U-ExM images showing enlarged views of centrioles, except for Fig. 5A and fig. S9 (A and B), used single slice images. Z-projections and brightness-contrast adjustments were made with the ImageJ software (version 1.8.0). For intensity quantification, the rough area was manually determined for each population in the images generated by maximum intensity Z-projections, and the largest mean intensity among all possible circles with diameters of 20 pixels (U-ExM) or 7 pixels (IF) throughout the area was used as the representative value. Twenty pixels in U-ExM and 7 pixels in IF correspond to 301 to 326 and 457 nm, respectively.

### Transmission electron microscopy

TEM for mouse male germ cells was performed as previously described (*57*, *58*). Seminiferous tubules were isolated from 7- to 14-week-old male mouse testes and then fixed with glutaraldehyde in cacodylate buffer [50 mM cacodylate (pH 7.2), 2.5% glutaraldehyde, 50 mM KCl, and 2.5 mM MgCl_2_] for 1 hour at 4°C. The fixed samples were washed five times with cacodylate buffer [50 mM cacodylate (pH 7.2), 50 mM KCl, and 2.5 mM MgCl_2_] for 3 min and then incubated with 2% osmium tetroxide in cacodylate buffer for 2 hours at 4°C. After washing with H_2_O three times, the samples were contrasted with 0.5% uranyl acetate solution overnight at 4°C. The samples were then dehydrated in an increasing ethanol series and incubated three times with propylene oxide for 30 min at room temperature. Subsequently, the samples were embedded in epon. Ultrathin sections (65 to 100 nm) were transferred to Formvar-coated copper grids and treated with 2% uranyl acetate solution for 20 min at room temperature. After a brief wash with H_2_O, the sections were counterstained with Reynold’s lead citrate for 10 min, washed with H_2_O, and dried at room temperature. The samples were analyzed with a JEOL JEM-2100 Transmission Electron Microscope (JEOL) equipped with an Olympus Veleta 2000 × 2000 camera system (JEOL) operated at 200 kV.

### Immunoprecipitation

Testes dissected from male mice were suspended in extraction buffer [20 mM tris-HCl (pH 7.5), 50 mM KCl, 5 mM MgCl_2_, 10% glycerol, 0.05% Triton X-100, and 1 mM β-mercaptoethanol] supplemented with cOmplete Protease Inhibitor (Roche, 5056489001) and Phosphatase Inhibitor (Roche, 4906837001). After homogenization, the extract was centrifuged at 16,500*g* for 45 min at 4°C. The supernatant was incubated with Dynabeads protein A (Thermo Fisher Scientific, 10002D) conjugated with antibodies or control IgG for 2 hours at 4°C. The beads were washed at least five times with high-salt buffer [20 mM Hepes (pH 7.0), 400 mM KCl, 5 mM MgCl_2_, 10% glycerol, 0.1% Triton X-100, and 1 mM β-mercaptoethanol], and the samples were eluted with 0.1 M glycine (pH 2.5).

### Immunoblotting

Protein samples in lithium dodecyl sulfate sample buffer (Thermo Fisher Scientific, NP0007) supplemented with 100 mM dithiothreitol were subjected to SDS–polyacrylamide gel electrophoresis on a precast 4 to 12% Bis-tris gel (Thermo Fisher Scientific, NW04122BOX) or a self-made 10% polyacrylamide gel and subsequently transferred to a nitrocellulose membrane (Bio-Rad, 1620094). The membrane was blocked with 5% skim milk in tris-buffered saline (TBS) containing 0.1% Tween (TBS-T) for 60 min at room temperature and then washed three times with TBS-T. The membrane was incubated with primary antibodies in 5% BSA in TBS-T for 2 hours at room temperature. After washing three times with TBS-T, the membrane was incubated with secondary antibodies in 5% skim milk in TBS-T for 1 hour at room temperature. The membrane was washed three times with TBS-T again before chemiluminescence detection. Signal detection was carried out with Amersham ECL Prime Western Blotting Detection Reagent (Cytiva, RPN2236) and ImageQuant LAS 500 (Cytiva).

### AlphaFold-Multimer prediction

The structural models of protein complexes were generated by AlphaFold-Multimer (*34*) using MMseq2 on ColabFold (*35*) (version 1.5.5) with the following settings: num_relax: 1, template_mode: none, msa_mode: mmseq2_uniref_env, pair_mode: unpaired_paired, model_type: auto, num_recycles: 3, recycle_early_stop_tolerance: auto, relax_max_iterations: 200, pairing_strategy: greedy, max_msa: auto, and num_seeds: 1. The representative images show the relaxed structural models that ranked first among five models, which were modified and exported by UCSF Chimera (version 1.17.3).

### Left-right axis assay

The left-right axes of the pups were determined by milk spots that are normally located on the left side. The left-right axes of the adult mice were determined by the position of heart that is normally located on the left side.

### Histological analysis

Testes and epididymides from male mice were fixed in Bouin’s fixative (Sigma-Aldrich, HT10132-1L) for 24 hours at room temperature and embedded in paraffin blocks. Slices of 8-μm thickness were stained with hematoxylin (Muto Pure Chemicals, 30002) and eosin (Muto Pure Chemicals, 32042). The stained slices were observed using a BX53 microscope (Olympus) with U-HGLGPS (Olympus) and U-TV1XC (Olympus).

### Fertility assay

After sexual maturation, one male mouse and one female mouse pair were placed together in one cage for a period of at least five months. During this period, the number of pups was counted at birth.

### Statistical analysis

Quantitative data are presented as the mean ± SD. Statistical analyses of the data were conducted using GraphPad Prism (version 10.4.1) and Microsoft Office Excel (version 2412). *P* values were determined by the appropriate tests as described in the respective figure legends. Each experiment showing only representative images was repeated independently at least twice with similar results.
