# Supplementary material for: Centrin-POC5 inner scaffold provides distal centriole integrity for sperm flagellar assembly
Source: Sci Adv. 2025 Dec 3;11(49):eaea4045. doi: 10.1126/sciadv.aea4045 (PMC12674131; doi:10.1126/sciadv.aea4045)
Supplement: Supplementary file 1 — Figs. S1 to S10 [file sciadv.aea4045_sm.pdf]

Science Advances
AAAS

Supplementary Materials for

Centrin-POC5 inner scaffold provides distal centriole integrity for sperm flagellar assembly

Yutaka Takeda *et al.*

Corresponding author: Hiroki Shibuya, hiroki.shibuya@riken.jp

Sci. Adv. **11**, eaea4045 (2025)
DOI: 10.1126/sciadv.aea4045

This PDF file includes:

Figs. S1 to S10

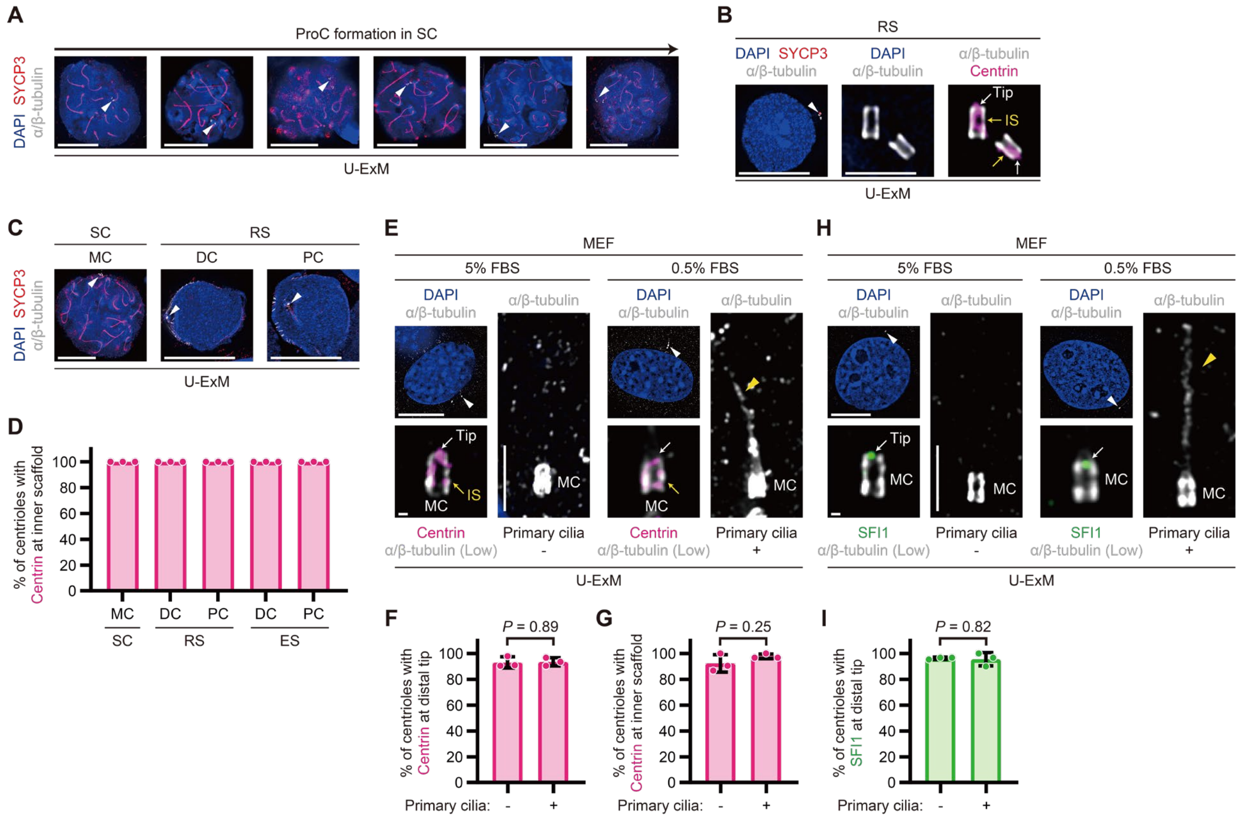

Fig. S1. Removal of distal tip proteins does not occur for ciliary assembly in somatic cells. (**A**) Uncropped U-ExM images corresponding to Figure 1D. Arrowheads: centrioles. SC: spermatocyte, ProC: procentriole. Scale bars: 10 µm. (**B**) U-ExM images of a round spermatid (RS) from WT male mice. Arrowhead: centrioles. Tip: distal tip, IS: inner scaffold. Scale bars: 10 µm and 1 µm. (**C**) Uncropped U-ExM images corresponding to Figure 2D. Arrowheads: centrioles. MC: mother centriole, DC: distal centriole, PC: proximal centriole. Scale bars: 10 µm. (**D**) Quantification of the frequency of centrioles with centrin signal at the inner scaffold in U-ExM images. *N* = 3 independent experiments with >30 cells each. (**E**) U-ExM images of WT mouse embryonic fibroblasts (MEFs) cultured in 5% FBS (control) or 0.5% FBS (serum-starved). Arrowheads: centrioles (white) and primary cilia (yellow). Scale bars: 10 µm, 1 µm, and 100 nm. (**F** and **G**) Quantification of the frequency of centrioles with centrin signal at the distal tip (F) and the inner scaffold (G) in U-ExM images. *N* = 3 independent experiments with >30 cells each. (**H**) U-ExM images of WT MEFs cultured in 5% FBS (control) or 0.5% FBS (serum-starved). Arrowheads: centrioles (white) and primary cilia (yellow). Scale bars: 10 µm, 1 µm, and 100 nm. (**I**) Quantification of the frequency of centrioles with SFI1 signal at the distal tip in U-ExM images. *N* = 3 independent experiments with >30 cells each. Data are presented as the mean ± s.d. *P* values were calculated by two-tailed unpaired Student's *t*-test (F, G, and I).

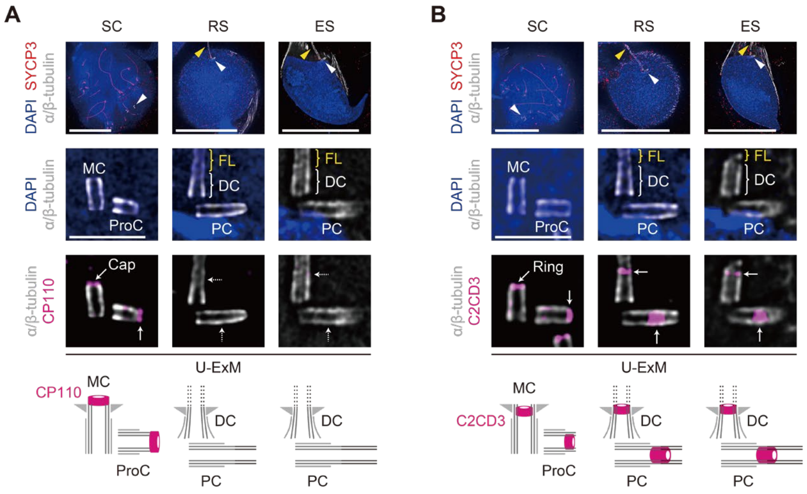

Fig. S2. The distal cap protein CP110 is removed during centriole transformations while the luminal distal ring protein C2CD3 is retained.

U-ExM images of spermatocytes (SCs), round spermatids (RSs), and elongating spermatids (ESs) from WT male mice. Arrowheads: centrioles (white) and flagella (yellow). MC: mother centriole, ProC: procentriole, FL: flagellum, DC: distal centriole, PC: proximal centriole, Cap: distal cap, Ring: luminal distal ring. Scale bars: 10 µm and 1 µm.

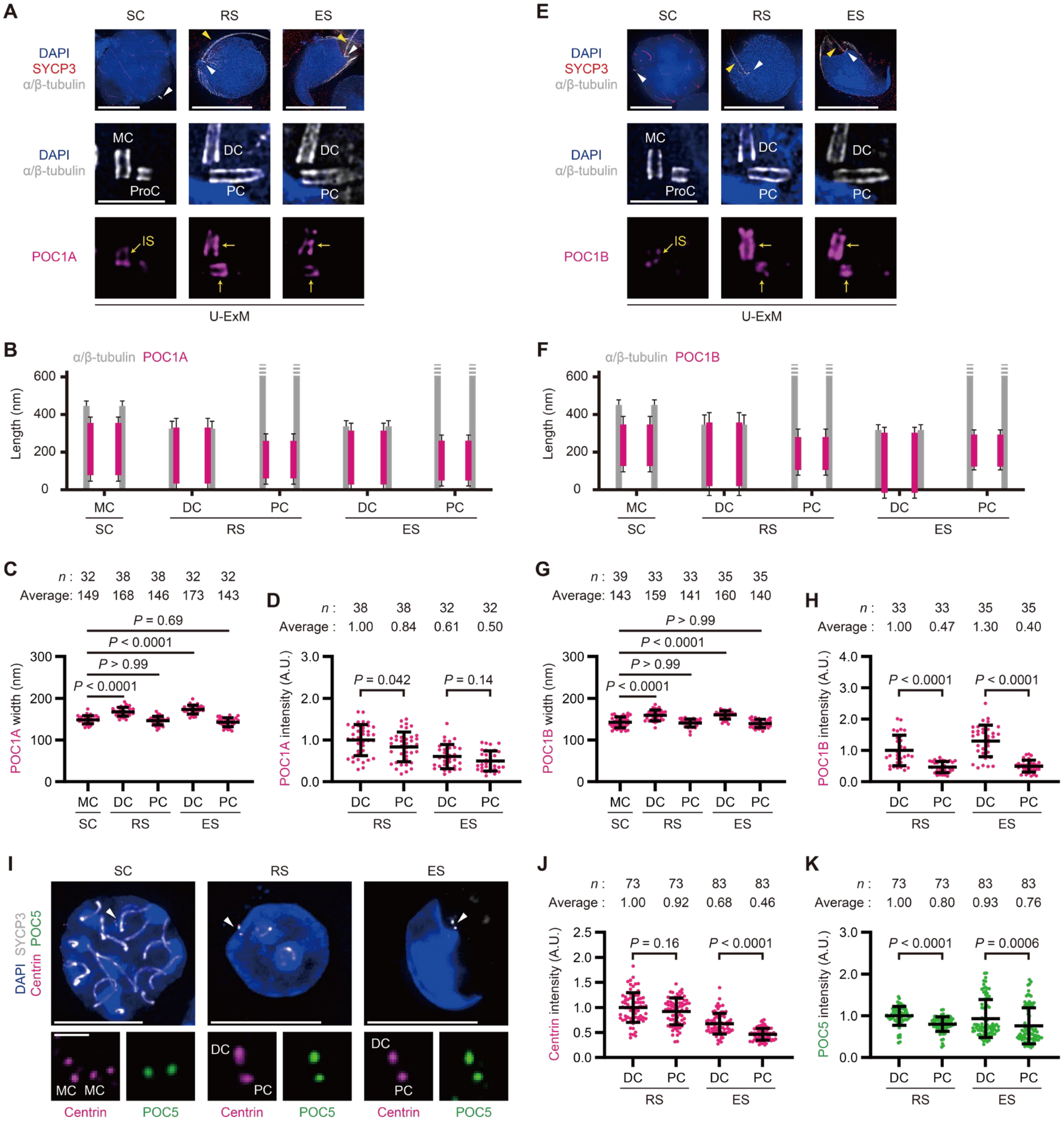

Fig. S3. The inner scaffold is augmented in the distal centriole during centriole transformations.

(**A**) U-ExM images of spermatocyte (SC), round spermatid (RS), and elongating spermatid (ES) from WT male mice. Arrowheads: centrioles (white) and flagella (yellow). MC: mother centriole, ProC: procentriole, FL: flagellum, DC: distal centriole, PC: proximal centriole, IS: inner scaffold. Scale bars: 10 µm and 1 µm. (**B**) Schematic showing the longitudinal positions of POC1A (magenta) relative to the α/β-tubulin signal (gray) calculated from >30 centrioles in U-ExM images. (**C**) Quantification of the widths of POC1A signals at centrioles in U-ExM images. (**D**) Quantification of POC1A intensity at centrioles in U-ExM images. (**E**) U-ExM images of SC, RS, and ES from WT male mice. Arrowheads: centrioles (white) and flagella (yellow). Scale bars: 10

μm and 1 μm. (**F**) Schematic showing the longitudinal positions of POC1B (magenta) relative to the α/β-tubulin signal (gray) calculated from >30 centrioles in U-ExM images. (**G**) Quantification of the widths of POC1B signals at centrioles in U-ExM images. (**H**) Quantification of POC1B intensity at centrioles in U-ExM images. (**I**) IF images of SC, RS, and ES from WT male mice. Arrowheads: centrioles. Scale bars: 10 μm and 1 μm. (**J** and **K**) Quantification of centrin (J) and POC5 (K) intensity at centrioles. Data are presented as the mean ± s.d. *P* values were calculated by one-way ANOVA with Dunn's multiple comparisons test (C and G) or Mann–Whitney *U* test (D, H, J, and K).

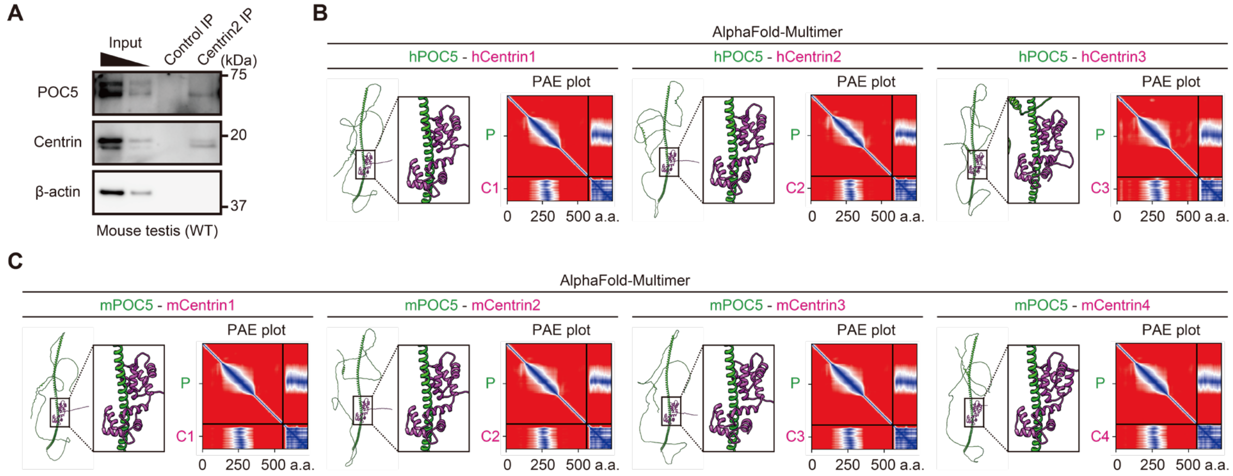

Fig. S4. POC5 interacts with centrin.

(**A**) Immunoblotting images of immunoprecipitated lysates from WT mouse testis extracts. (**B** and **C**) Structural model and predicted aligned error (PAE) plot of the protein complex of POC5 and each centrin paralog in humans (B) and mice (C) generated by AlphaFold-Multimer.

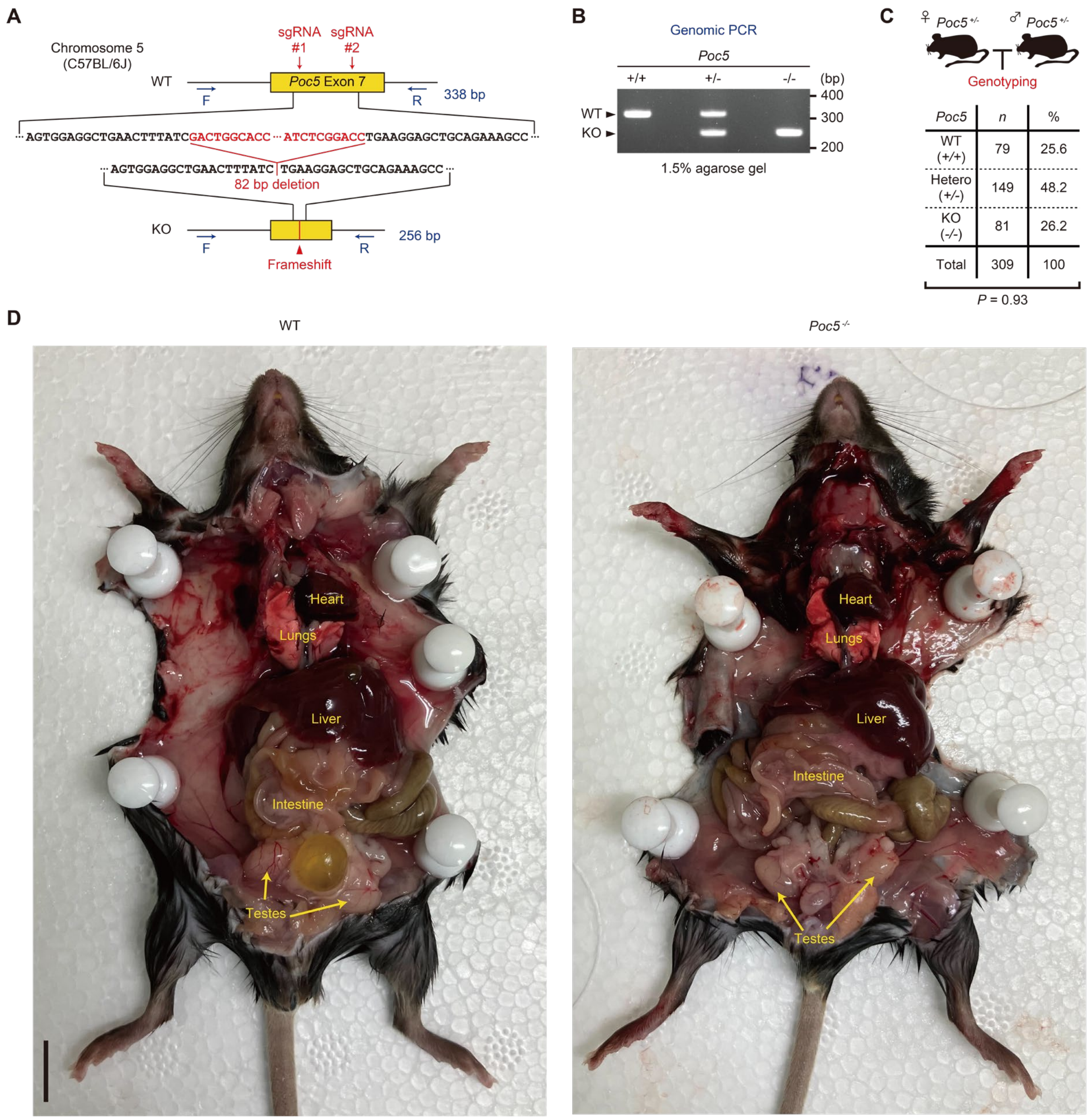

Fig. S5. POC5 is dispensable for mouse development.

(**A**) Schematic of CRISPR-mediated knockout of *Poc5* in mice. (**B**) Genomic PCR and agarose gel electrophoresis using mouse tails. (**C**) Genotyping results of pups from *Poc5*$^{+/-}$ mouse pairs. *N* = 27 litters. (**D**) Images of dissected mice. Scale bar: 1 cm. *P* value was calculated by chi-square test (C).

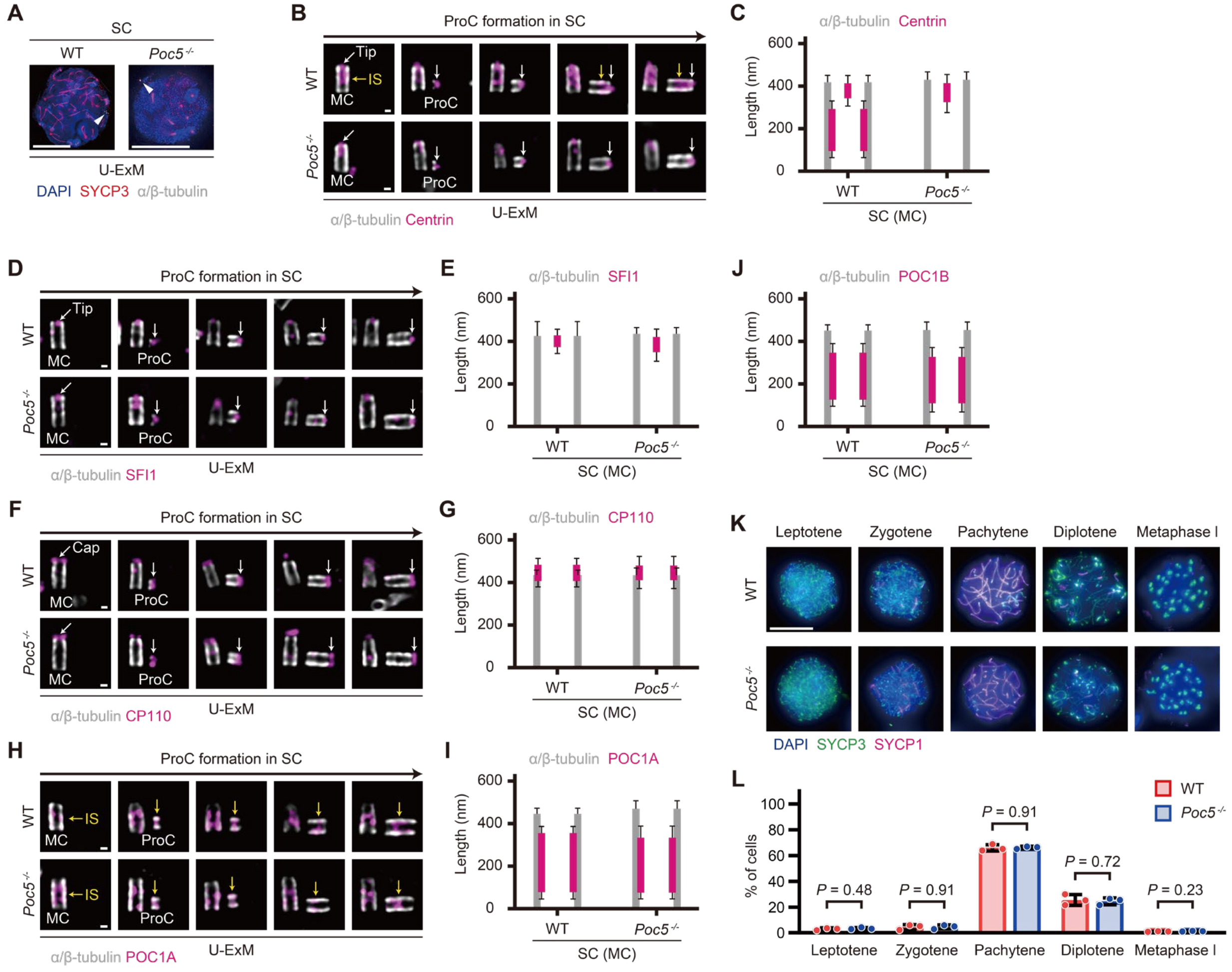

Fig. S6. POC5 is dispensable for centriole integrity and function in spermatocytes.

(**A**) Uncropped U-ExM images corresponding to Figure 4J. Arrowheads: centrioles. SC: spermatocyte. Scale bars: 10 µm. (**B**) U-ExM images of SCs from male mice. Images of different SCs were arranged according to the length of the procentrioles (ProCs). MC: mother centriole, Tip: distal tip, IS: inner scaffold. Scale bars: 100 nm. (**C**) Schematic showing the longitudinal positions of centrin (magenta) relative to the α/β-tubulin signal (grey) calculated from >30 MCs in U-ExM images of SCs. WT is identical to MC/SC in Figure 2C. (**D**) U-ExM images of SCs from male mice. Images of different SCs were arranged according to the length of the ProCs. Scale bars: 100 nm. (**E**) Schematic showing the longitudinal positions of SFI1 (magenta) relative to the α/β-tubulin signal (grey) calculated from >30 MCs in U-ExM images of SCs. (**F**) U-ExM images of SCs from male mice. Images of different SCs were arranged according to the length of the ProCs. Cap: distal cap. Scale bars: 100 nm. (**G**) Schematic showing the longitudinal positions of CP110 (magenta) relative to the α/β-tubulin signal (grey) calculated from >30 MCs in U-ExM images of SCs. (**H**) U-ExM images of SCs from male mice. Images of different SCs were arranged according to the length of the ProCs. Scale bars: 100 nm. (**I**) Schematic showing the longitudinal positions of POC1A (magenta) relative to the α/β-tubulin signal (grey) calculated from >30 MCs in U-ExM images of SCs. WT is identical to MC/SC in Figure S3B. (**J**) Schematic showing the longitudinal positions of POC1B (magenta) relative to the α/β-tubulin signal (grey) calculated from

>30 MCs in U-ExM images of SCs. WT is identical to MC/SC in Figure S3F. (**K**) IF images of SCs from 12-week-old male mice. The cells were classified into the substages based on the SYCP3 and SYCP1 signals. Scale bar: 10 µm. (**L**) Quantification of the frequency of cells classified into each substage. $N = 3$ independent experiments with >500 cells each. Data are presented as the mean ± s.d. P values were calculated by two-tailed unpaired Student's t-test (L).

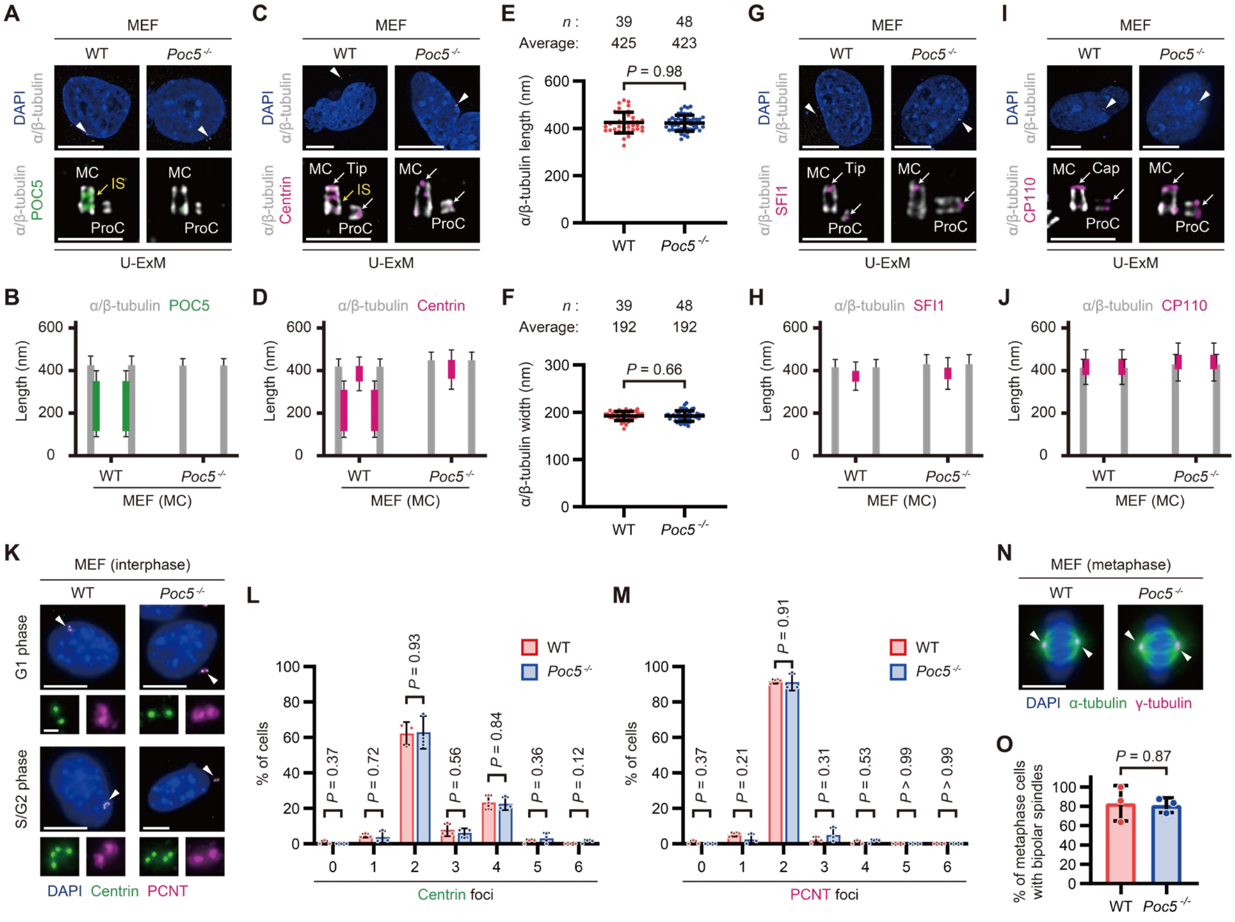

Fig. S7. POC5 is dispensable for centriole integrity and function in somatic cells.

(**A**) U-ExM images of mouse embryonic fibroblasts (MEFs). Arrowheads: centrioles. MC: mother centriole, ProC: procentriole, IS: inner scaffold. Scale bars: 10 µm and 1 µm. (**B**) Schematic showing the longitudinal positions of POC5 (green) relative to the α/β-tubulin signal (grey) calculated from >30 MCs in U-ExM images of MEFs. (**C**) U-ExM images of MEFs. Arrowheads: centrioles. Tip: distal tip. Scale bars: 10 µm and 1 µm. (**D**) Schematic showing the longitudinal positions of centrin (magenta) relative to the α/β-tubulin signal (grey) calculated from >30 MCs in U-ExM images of MEFs. (**E** and **F**) Quantification of the MC length (E) and width (F) based on the α/β-tubulin signal in U-ExM images of MEFs. (**G**) U-ExM images of MEFs. Arrowheads: centrioles. Scale bars: 10 µm and 1 µm. (**H**) Schematic showing the longitudinal positions of SFI1 (magenta) relative to the α/β-tubulin signal (grey) calculated from >30 MCs in U-ExM images of MEFs. (**I**) U-ExM images of MEFs. Arrowheads: centrioles. Cap: distal cap. Scale bars: 10 µm and 1 µm. (**J**) Schematic showing the longitudinal positions of CP110 (magenta) relative to the α/β-tubulin signal (grey) calculated from >30 MCs in U-ExM images of MEFs. (**K**) IF images of MEFs in interphase. The interphase cells were roughly classified into the G1 and the S/G2 phase based on the number of centrin foci. Arrowheads: centrioles. Scale bars: 10 µm and 1 µm. (**L** and **M**) Quantification of the frequency of interphase cells with the indicated number of centrin (L) and PCNT (M) foci. *N* = 3 independent experiments with >50 cells each. (**N**) IF images of MEFs in metaphase. Arrowheads: spindle poles. Scale bar: 10 µm. (**O**) Quantification of the frequency

of metaphase cells with bipolar spindles. $N = 3$ independent experiments with >10 cells each. Data are presented as the mean ± s.d. P values were calculated by Mann–Whitney U test (E and F) or two-tailed unpaired Student's t-test (L, M, and O).

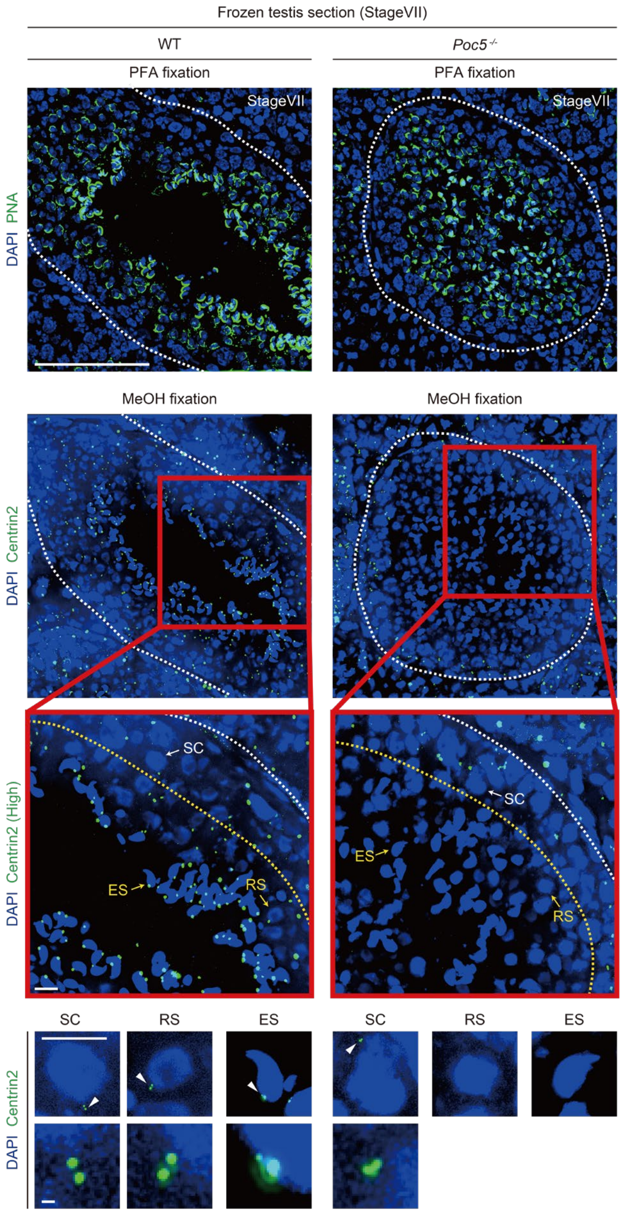

Fig. S8. POC5 is required for the localization of centrin at centrioles in spermatids.

IF images of frozen testis sections from male mice. Frozen sections of the same seminiferous tubule are shown for each genotype. Stages were determined using DAPI and PNA signals in the paraformaldehyde (PFA)-fixed sections. Arrowheads: centrioles, dotted lines: periphery of seminiferous tubules (white) and boundary between the spermatocyte layer and the spermatid layer (yellow). MeOH: methanol, SC: spermatocyte, RS: round spermatid, ES: elongating spermatid. Scale bars: 100 µm, 10 µm, 10 µm, and 1 µm.

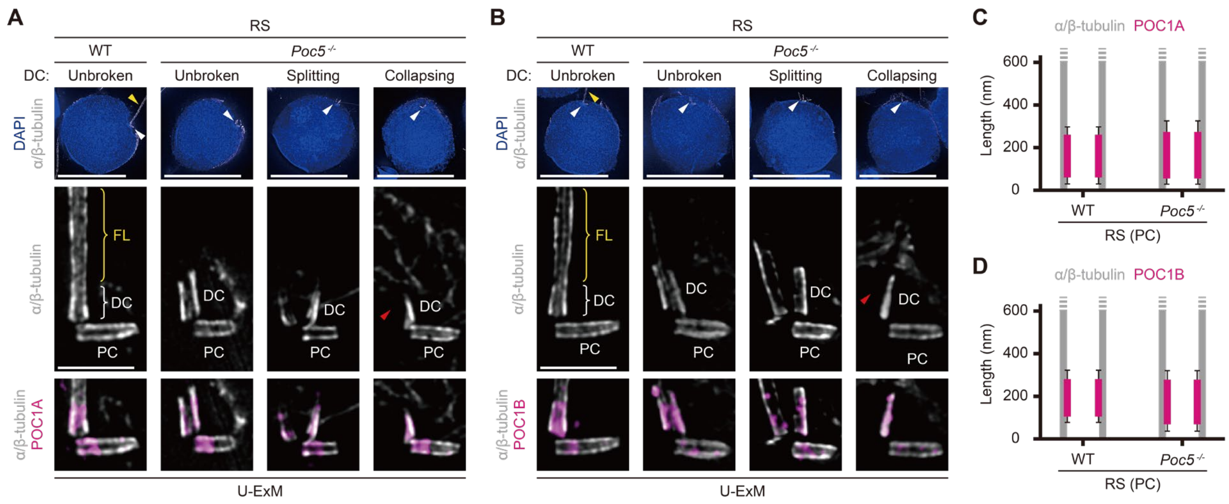

Fig. S9. POC1A and POC1B show structural disruption at distal centrioles, but not at proximal centrioles, in *Poc5* KO spermatids.

(**A** and **B**) U-ExM images of round spermatids (RSs) from male mice. Arrowheads: centrioles (white), flagellum (yellow), and missing centriole wall (red). FL: flagellum, DC: distal centriole, PC: proximal centriole. Scale bars: 10 µm and 1 µm. (**C** and **D**) Schematic showing the longitudinal positions of POC1A (magenta, C) and POC1B (magenta, D) relative to the α/β-tubulin signal (grey) calculated from >30 PCs in U-ExM images of RSs. WT is identical to PC/RS in Figure S3B (C) or S3F (D).

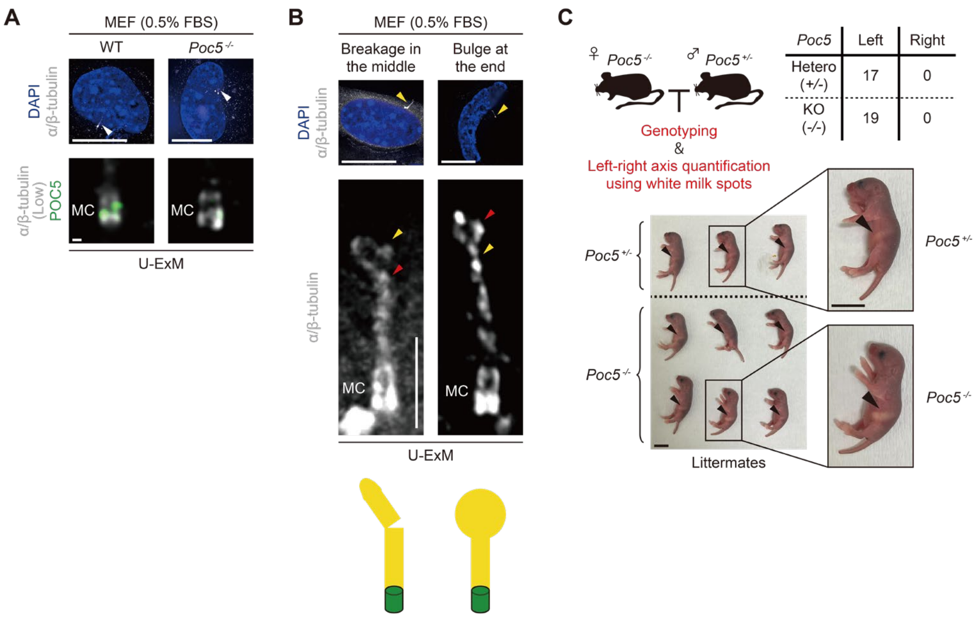

Poc5	Left	Right
Hetero (+/-)	17	0
KO (-/-)	19	0

Fig. S10. POC5 is dispensable for ciliary assembly in somatic cells.

(**A**) Supplementary U-ExM images related to Figure 5K. Arrowheads: primary cilia. MC: mother centriole. Scale bars: 10 μm and 100 nm. (**B**) Supplementary U-ExM images related to Figure 5L. Examples of defective primary cilia in the quantification. Arrowheads: primary cilia (yellow) and defective sites (red). Scale bars: 10 μm and 1 μm. (**C**) Quantification and representative images of the stomach positions in pups from *Poc5*$^{-/-}$ and *Poc5*$^{+/-}$ mouse pairs with their genotyping results. The stomach position was determined by a white milk spot for each pup. Arrowheads: stomachs. Scale bars: 1 cm. $N = 5$ litters.
